# Linking single-cell measurements of mass, growth rate, and gene expression

**DOI:** 10.1186/s13059-018-1576-0

**Published:** 2018-11-27

**Authors:** Robert J. Kimmerling, Sanjay M. Prakadan, Alejandro J. Gupta, Nicholas L. Calistri, Mark M. Stevens, Selim Olcum, Nathan Cermak, Riley S. Drake, Kristine Pelton, Frederik De Smet, Keith L. Ligon, Alex K. Shalek, Scott R. Manalis

**Affiliations:** 1 Koch Institute for Integrative Cancer Research, Massachusetts Institute of Technology, Cambridge, MA 02139 USA; 2 Department of Biological Engineering, Massachusetts Institute of Technology, Cambridge, MA 02139 USA; 3 Ragon Institute of Massachusetts General Hospital, Massachusetts Institute of Technology, and Harvard, Cambridge, MA 02139 USA; 4 Department of Chemistry, Massachusetts Institute of Technology, Cambridge, MA 02139 USA; 5 Institute for Medical Engineering & Science, Massachusetts Institute of Technology, Cambridge, MA 02139 USA; 6 Broad Institute of MIT and Harvard, Cambridge, MA 02142 USA; 7 Department of Medical Oncology, Dana-Farber Cancer Institute, Harvard Medical School, Boston, MA 02215 USA; 8 Department of Oncologic Pathology, Dana-Farber Cancer Institute, Boston, MA 02215 USA; 9 Department of Imaging and Pathology, KU Leuven, Leuven, Belgium; 10 Harvard-MIT Division of Health Sciences and Technology, Massachusetts Institute of Technology, Cambridge, MA 02139 USA; 11 Massachusetts General Hospital, Boston, MA 02114 USA; 12 Department of Mechanical Engineering, Massachusetts Institute of Technology, Cambridge, MA 02139 USA

**Keywords:** Single-cell RNA-Seq, Mass, Growth, Serial suspended microchannel resonator, Multi-omics, Single cell, T cell activation, Glioblastoma, GBM, Drug response, Microfluidics, Biophysical properties

## Abstract

Mass and growth rate are highly integrative measures of cell physiology not discernable via genomic measurements. Here, we introduce a microfluidic platform enabling direct measurement of single-cell mass and growth rate upstream of highly multiplexed single-cell profiling such as single-cell RNA sequencing. We resolve transcriptional signatures associated with single-cell mass and growth rate in L1210 and FL5.12 cell lines and activated CD8+ T cells. Further, we demonstrate a framework using these linked measurements to characterize biophysical heterogeneity in a patient-derived glioblastoma cell line with and without drug treatment. Our results highlight the value of coupled phenotypic metrics in guiding single-cell genomics.

**Electronic supplementary material:**

The online version of this article (10.1186/s13059-018-1576-0) contains supplementary material, which is available to authorized users.

## Background

Recent experimental advancements have dramatically improved the throughput and cost-efficiency of single-cell RNA sequencing (scRNA-seq) [1–3]. However, gene expression measurements alone cannot fully describe many complex cellular processes [4, 5]. Thus, parallel efforts have focused on linking single-cell transcriptomics with complementary data that can provide further information to help guide analyses and contextualize distinct cellular states. For instance, various multi-omic methods have been developed to link measurements such as protein abundance, DNA sequence, or methylation with gene expression from the same single cell [6–9]. Gene expression measurements have also been linked to single-cell location within a tissue to enable study of cellular development and differentiation at unprecedented detail [10–12]. Moreover, single-cell functional assays have been coupled with mRNA expression to obtain novel insights into the relationships among cellular electrophysiology, morphology, and transcription [13]. Taken together, these approaches demonstrate how linked single-cell data sets can afford a deep understanding of various cellular phenotypic states that may be difficult to glean through transcriptomic measurements alone.

Linked gene expression data sets are of particular interest when considering recent technological developments that enable the precise measurement of various single-cell biophysical properties, such as mass and growth rate [14, 15]. As highly integrative metrics of cellular state, these parameters offer unique insights into a wide range of biological phenomena, including (i) basic patterns of single-cell mass and growth regulation; (ii) biophysical changes associated with immune cell activation; and, (iii) cancer cell heterogeneity in the presence or absence of drug [16–18]. However, the approaches and devices previously used to collect these biophysical measurements have precluded linking these properties with molecular profiling of the same cell.

To our knowledge, there have been no methods reported to date that allow for linked measurements of cellular mass, growth rate, and transcriptome-wide gene expression from the same cell. It has therefore been challenging to characterize the underlying transcriptional programs associated with cellular mass and growth rate variability observed in a range of normal and dysfunctional biological contexts.

Here, we describe and characterize a microfluidic platform that enables the measurement of single-cell mass and growth rate immediately upstream of a range of highly multiplexed single-cell endpoint assays. We leverage this approach in combination with scRNA-seq to examine linked single-cell biophysical and transcriptomic properties in cell lines and primary cells. Finally, we apply this method to examine biophysical heterogeneity in a patient-derived glioblastoma (GBM) cancer cell line in the presence or absence of drug, highlighting the potential utility of guiding single-cell genomic measurements with biophysical metadata.

## Results and discussion

### Serial SMR platform with downstream collection for scRNA-seq

**Fig. 1:**
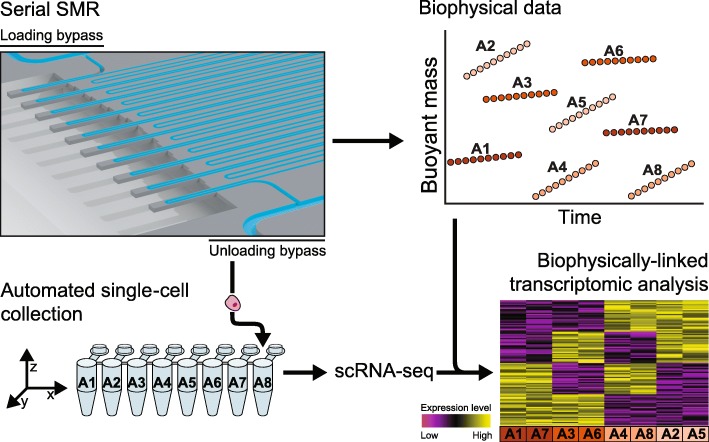
Serial SMR platform with downstream collection for scRNA-seq. Schematic representation of the serial SMR platform, which includes an array of SMR mass sensors, separated by a serpentine delay channel to periodically measure the buoyant mass of a single cell. Independent control of the upstream and downstream pressures applied to two bypass channels allows for single-cell spacing at the loading entrance of the array (top left of sSMR image) and single-cell isolation at the unloading exit (bottom right of sSMR image) (Additional file 1: Figure S1, Additional file 1: Note S1). Using real-time peak detection at the final mass sensor, a three-dimensional motorized stage is triggered to capture each individual cell directly in lysis buffer for downstream scRNA-seq. Based on well location each cell is subsequently matched to its corresponding biophysical data collected from the sSMR, including mass and MAR, as schematized in the top-right panel. These linked single-cell data sets can then be used to determine gene expression signatures associated with mass and growth rate variability, as schematized in the bottom-right panel

Our system relies on a modified version of a previously described serial suspended microchannel resonator (sSMR) device (Fig. 1) that utilizes an array of high-resolution single-cell buoyant mass sensors placed periodically along the length of a long microfluidic channel to allow a single cell’s mass to be measured periodically as it traverses the channel [17]. In addition to providing mass information, this series of measurements can also be used to determine the mass accumulation rate (MAR), or growth rate, of each cell. Here, taking advantage of real-time access to the data generated by each SMR mass sensor, we have modified the system to use peak detection in the final cantilever. Detection at this cantilever indicates a cell exiting the mass sensor array and triggers the motion of a three-dimensional motorized stage which positions a PCR tube containing lysis buffer to capture each single cell as it is flushed from the system. This enables, for the first time, measurements of the biophysical properties of mass and growth rate to be linked to genomic profiles—here RNA-seq—at the single-cell level (Methods).

We sought to endow our platform with sufficiently high throughput to enable measurements on populations of cells that may change over time. The total time required to flush the system’s dead volume and release each single cell (20 s for the system implementation described here) sets a theoretical maximum throughput for the platform to avoid the collection of multiplets. Crucially, to minimize the frequency of failed capture events, we implemented a new fluidic scheme whereby single cells are loaded into the array of mass sensors at fixed intervals (Additional file 1**:** Figure S1, Additional file 1: Note S1) [19]. Ultimately, this fluidic scheme allows us to achieve a throughput of one cell approximately every 30 s (for a throughput of up to 120 cells per hour) with minimal failed collection events due to co-release. This offers a two-fold throughput improvement over previous implementations of biophysical measurements alone, while affording the additional ability to capture each individual cell downstream for processing—e.g., scRNA-seq.

### Unique gene expression profiles related to specific biophysical properties and underlying cell biology

**Fig. 2:**
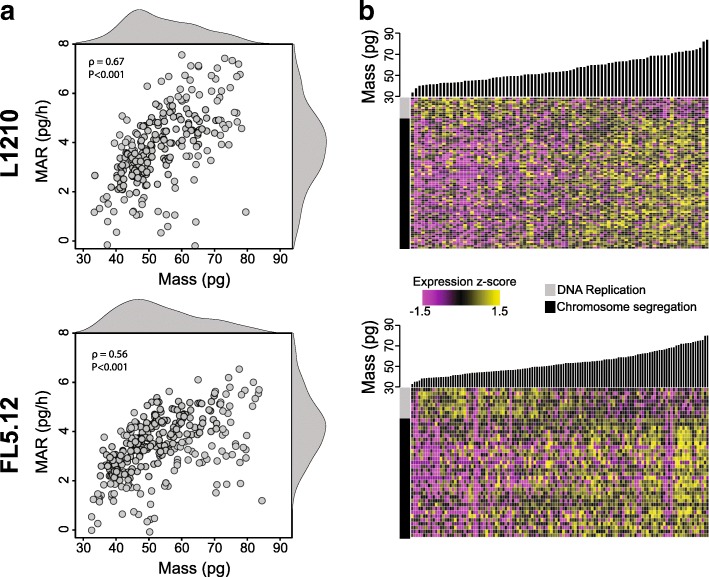
Linked biophysical and gene expression measurements of single L1210 and FL5.12 cells. **a** Plot of mass accumulation rate versus buoyant mass for single L1210 cells (top, *n* = 234) and single FL5.12 cells (bottom, *n* = 296) measured in the sSMR. Kernel density plots are included on both axes. **b** Heat maps showing the relative expression of various cell cycle-related genes for subsets of the L1210 (top, *n* = 85) and FL5.12 (bottom, *n* = 124) cells depicted in (**a**) that were captured downstream for scRNA-seq. Cells are ordered by buoyant mass (bar plots above heat maps). Entries are colored by expression *z*-score. As a demonstration, the heat map includes genes with expression levels that showed a significant correlation with buoyant mass from the chromosome segregation (black bar, *n* = 58 and *n* = 31 for the L1210 and FL5.12, respectively) and DNA replication (gray bar, *n* = 11 and n = 8 for the L1210 and FL5.12, respectively) gene ontology subsets (FDR < 0.05, Additional file 1: Figure S4, Additional file 3: Table S2, Additional file 4: Table S3, Methods)

To validate our method for collecting linked single-cell biophysical and gene expression data, we first measured two murine lymphoblast cell lines (L1210 and FL5.12) that have well-characterized mass and growth properties that are stable over the course of long-term propagation in bulk culture (Fig. 2) [15–17, 20]. Single cells collected downstream of the sSMR for scRNA-seq consistently yielded high-quality cDNA libraries, with 85 out of 87 individual L1210 cells and 124 out 144 individual FL5.12 cells with paired biophysical data passing initial quality controls (e.g., number of genes detected greater than 4000, Methods, Additional file 1: Figure S2).

In order to determine the transcriptional signatures associated with the spectrum of biophysical states in these cells, we ranked genes by how strongly their expression levels correlated with single-cell biophysical data (Spearman’s correlation coefficients, Additional file 2: Table S1; NB Both Spearman and Pearson correlation methods yielded similar results for all comparisons considered, Additional file 1: Figure S3). We then utilized the GSEA Preranked tool to determine which gene sets showed significant enrichment at either end of these ranked lists (FDR < 0.05, Methods, Additional file 3: Table S2) [21]. For both cell lines, genes ranked by correlation strength with single-cell mass (final mass measurement collected before cell lysis) were highly enriched for functional annotations relating to cell cycle progression (FDR < 0.05, Additional file 3: Table S2, Fig. 2). Specifically, genes related to early cell cycle events immediately following cell division—such as DNA replication initiation—were more highly expressed in cells with lower masses, whereas genes related to late cell cycle events that occur just prior to division—such as chromosome segregation—were more highly expressed in cells with higher masses (Additional file 4: Table S3). Interestingly, both cell lines revealed a larger number of genes that showed a significant positive correlation with mass relative to the number of genes with a significant negative correlation, though this may be impacted, in part, by the transcript capture inefficiencies inherent in scRNA-seq protocols (Additional file 1: Figure S4) [22].

The manifestation of cell cycle-related gene expression in scRNA-seq data has been of particular interest to both further characterize mechanisms of cell cycle progression and regress out the contributions of cell cycle variability that may act as a nuisance parameter, dominating gene expression heterogeneity among single cells and masking more subtle biological signals [2, 23, 24]. We therefore sought to determine how previously annotated cell cycle signatures corresponded to the single-cell mass measurements collected here. To do so, we established cell cycle phase-specific (G1/S and G2/M) scores using an approach inspired by Macosko et al. [2] (Additional file 1: Figure S5, Additional file 1: Note S3). Cells assigned to either the G1/S or G2/M phases of the cell cycle using gene expression data alone showed significant differences in cell mass for both the L1210 and FL5.12 cells that were consistent with expectations (i.e., lower mass for G1/S cells; *P* < 0.001, Mann-Whitney *U* test). Furthermore, for both cell types, cell mass showed a clear negative correlation with G1/S scoring (*ρ* = − 0.46 and *ρ* = − 0.25 for L1210 and FL5.12, respectively; *P* < 0.005) and a clear positive correlation with G2/M scoring (*ρ* = 0.74 and *ρ* = 0.54 for L1210 and FL5.12, respectively; *P* < 0.001). Together, these results provide additional evidence of coordination between cell mass and cell cycle-related gene expression in actively proliferating cells.

To further confirm the consistency and reproducibility of the linked biophysical and gene expression data sets collected with this platform, we compared the L1210 and FL5.12 results with scRNA-seq data from additional independent experiments. For L1210 cells, we found that genes that showed significant correlations with cell mass were also significantly enriched among those previously shown to correlate with time since cell division, a proxy for cell cycle progression (FDR < 0.05, Additional file 1: Figure S6, Additional file 1: Note S2) [25]. In FL5.12 cells, meanwhile, we observed that the genes which showed significant correlations between their expression levels and biophysical properties were highly reproducible across two independent linked biophysical and gene expression experiments (FDR < 0.05, Additional file 1: Figure S6, Additional file 1: Note S2). These results demonstrate the quality and reproducibility of transcriptional measurements collected downstream of the sSMR.

Given that we identified a linear relationship between mass and MAR in these cell types (*ρ* = 0.67 and *ρ* = 0.56 for L1210 and FL5.12, respectively; *P* < 0.001, Fig. 2), we focused our analysis on mass-normalized MAR, determined by dividing each cell’s MAR by its corresponding mass. We used this parameter, which measures a single cell’s growth efficiency decoupled from mass-related confounders, to resolve growth-related transcriptional signatures in these two cell lines [18, 26]. For L1210 cells, genes ranked by strength of correlation between expression level and growth efficiency did not reveal any statistically significant enrichment of functional annotations (FDR > 0.05). The FL5.12 cells, however, showed significant positive enrichment for functional annotations related to cell cycle progression among genes ranked by correlation strength with growth efficiency (FDR < 0.05, Additional file 3: Table S2). Specifically, subsets of genes implicated in the G1-S transition showed a higher level of expression in cells of intermediate mass with the highest growth efficiencies (Methods, Additional file 1: Figure S7, Additional file 5 : Table S4) [27]. These results are consistent with previous FL5.12 single-cell growth measurements, which revealed an increase in growth efficiency approaching the G1-S transition followed by a decrease later in the cell cycle [15].

### Characterizing CD8+ T cell activation with linked biophysical and gene expression measurements

While the L1210 and FL5.12 cells represent effective model systems with stable biophysical and transcriptional profiles, one of the benefits of the sSMR platform is that it offers sufficient throughput to characterize cell populations that may be changing in their phenotypes over time [20, 25]. Primary CD8+ T lymphocytes are a prime example of a cell population that may exhibit dynamic behavior, as they are known to drastically change their biophysical properties, transcriptional states, and metabolic characteristics in response to activation [17, 28, 29].

**Fig. 3:**
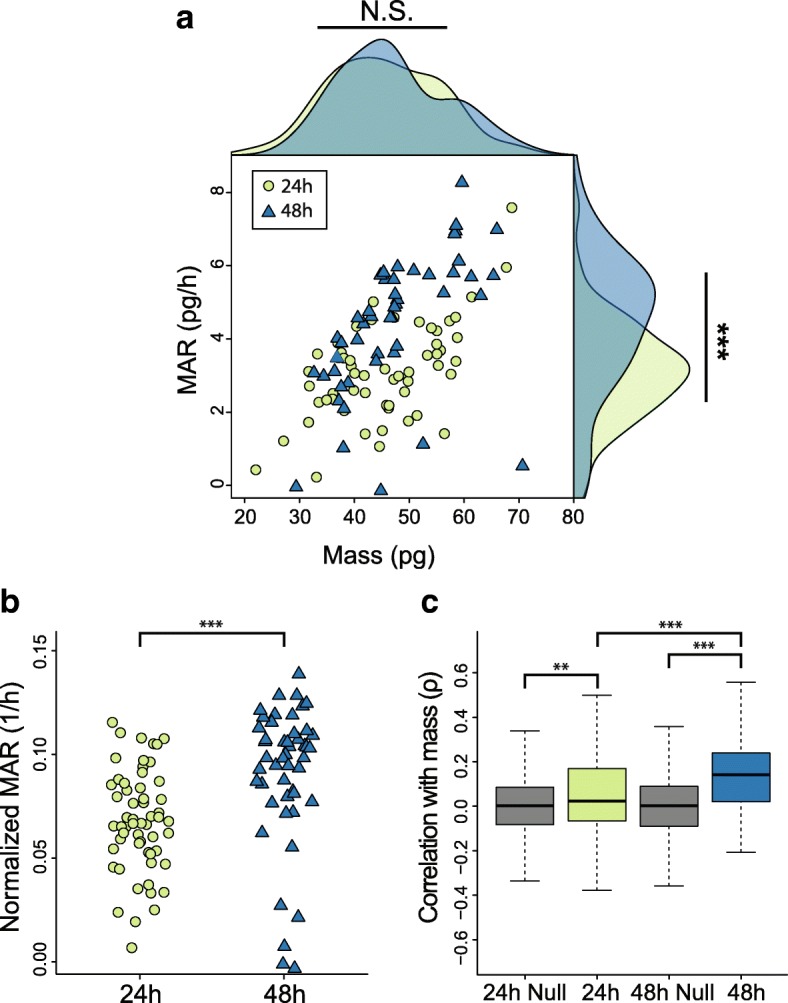
Linked biophysical and gene expression measurements of activated murine CD8+ T cells. **a** Plot of mass accumulation rate versus buoyant mass for murine CD8+ T cells after 24 h (green points, *n* = 59) or 48 h (blue triangles, *n* = 49) of activation in vitro. Kernel density plots, using the same color scheme, are included on the margins for both populations. ***P < 0.001, N.S. indicates not significant; Mann-Whitney *U* test. **b** Plot of mass-normalized single-cell growth rates (growth efficiency) for the same murine CD8+ T cells activated for 24 or 48 h in vitro. Groups were compared with a Mann-Whitney *U* test (***P < 0.001). **c** Box charts showing the Spearman correlation coefficients between single-cell mass measurements and the expression of a subset of genes previously found to be related to cell cycle in activated CD8+ T cells (300 genes) for cells activated for 24 or 48 h. For comparison, the null distribution of Spearman correlation coefficients for the same subset of cells after randomly assigning single-cell mass measurements is shown for each time point (gray boxes, Methods). Groups were compared with a Mann-Whitney *U* test (***P < 0.001, **P < 0.01)

To characterize this response, we collected single-cell biophysical and gene expression profiles from freshly isolated, naïve murine CD8+ T cells which we stimulated in vitro with antibody-based T cell receptor engagement and CD28 co-stimulation (Fig. 3, Methods). We chose to evaluate the 24 and 48 h time points to capture cells before and after their first division event, respectively [30]. Although the cells for both time points displayed similar mass distributions, the cells measured after 48 h of activation showed significantly higher growth efficiencies (*P* < 0.001, Mann-Whitney *U* test, Fig. 3a, b).

Examining gene expression alone, we observed that cells from these two populations showed differential expression patterns consistent with T cell activation, including significant upregulation of Granzyme B (*Gzmb*) and IL-2 receptor (*Il2ra* and *Il2rb*), as well as significant downregulation of *Ccr7* in the 48 h population compared to the 24 h one (Bonferroni-corrected *P* < 0.05, Additional file 6: Table S5). Similarly, gene set enrichment analysis performed on genes ranked by expression fold change between these time points revealed significant enrichment for gene sets related to immune cell effector function and glucose metabolism, consistent with functional and metabolic shifts that have been previously characterized in activated CD8+ T cells (FDR < 0.05, Additional file 7: Table S6, Additional file 8: Table S7) [28, 31]. Cells activated for 48 h also displayed a higher expression of genes related to protein production, including those involved in translation initiation and cytosolic ribosome activity (Additional file 8: Table S7). Araki et al. recently demonstrated a similar trend, noting an increase in translation activity over of the course of early T cell activation, as cells become more proliferative [32]. The measurements presented here suggest that this increase in translation activity is accompanied by, and potentially is tied to, increased growth efficiency observed at 48 h compared to 24 h.

This population-level relationship between growth efficiency and translation-related gene expression was also observable at the single-cell level for cells activated for 48 h. Within this time point, genes ranked by correlation strength with single-cell growth efficiency once again showed significant enrichment for functional annotations relating to translation machinery (FDR < 0.05, Additional file 3: Table S2). Despite a similar number of genes showing a significant correlation with growth efficiency at the 24 h time point, these genes did not show any significant functional enrichment when ranked by correlation strength (FDR > 0.05, Additional file 1: Figure S4). This result suggests that the coordination between single-cell growth efficiency and translation-related gene expression occurs later during T cell activation.

The 48 h time point also revealed a greater number of genes that showed a significant correlation between expression level and cell mass relative to the 24 h time point (Additional file 1: Figure S4). When determining the functional role of genes ranked by expression correlation with single-cell mass, the 48 h time point demonstrated significant cell cycle functional enrichment (FDR < 0.05) whereas the 24 h time point only showed a slight enrichment for similar cell cycle-related annotations (FDR < 0.1) and no significantly enriched terms otherwise (Additional file 3: Table S2). However, when conducting a cell cycle phase scoring analysis similar to that described for the L1210 and FL5.12 cells, we found that both the 24 and 48 h time points showed a significant difference in mass between cells assigned to the G1/S and G2/M phases of the cell cycle (*P* < 0.001, Mann-Whitney *U* test, Additional file 1: Figure S5). Furthermore, a previously described set of genes known to correlate with an activated CD8+ T cell’s time since division—a proxy for cell cycle progression—showed a significant positive correlation with cell mass in both the 24 h and 48 h populations, though the strength of this correlation did increase significantly by 48 h (*P* < 0.001, Mann-Whitney *U* test, Fig. 3) [25]. As mentioned above, the 24 and 48 h time points capture cells before and after their first division event, respectively [30]. Although cells are accumulating mass, or “blasting,” in the first 24 h, it is not until roughly 30 h that cells undergo their first division and begin increasing in number and cycling in the traditional sense [30, 33]. Taken together, these results suggest that the coordination between cell cycle gene expression and cell mass begins early during T cell activation, even before cells begin proliferating, and increases in strength later in T cell activation as cells begin actively dividing.

### Characterizing single-cell biophysical heterogeneity of a patient-derived cancer cell line

**Fig. 4:**
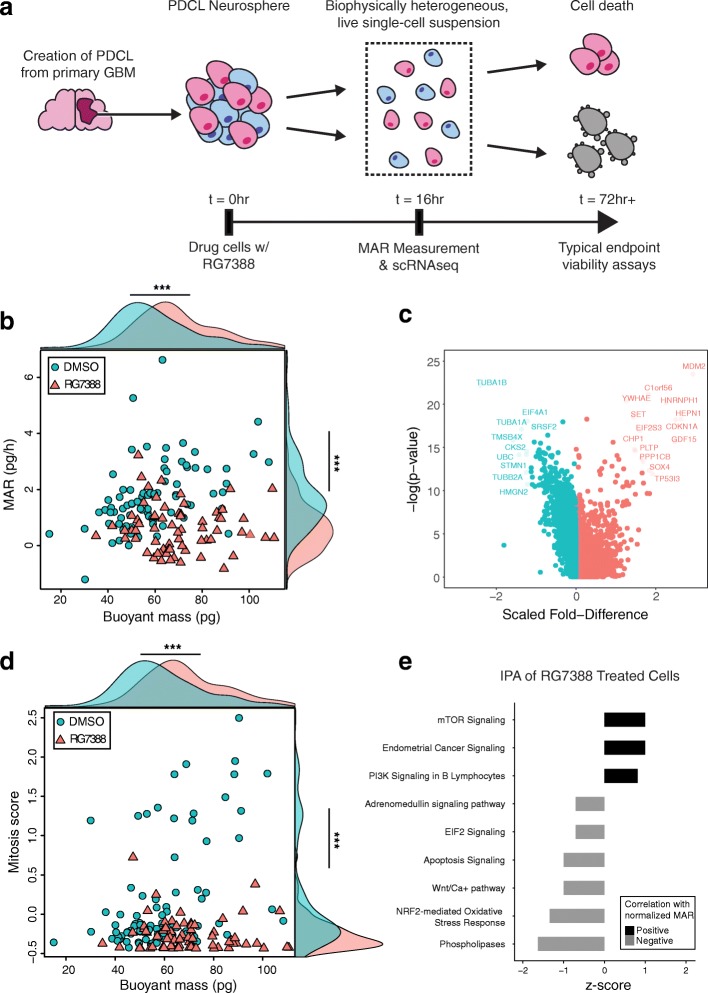
Characterizing single-cell drug response in BT159 GBM cells. **a** Schematic representation of GBM PDCL generation, drug treatment in vitro, and subsequent characterization of therapeutic response using the sSMR collection platform. Mass and growth measurements are collected after 16 h of treatment, prior to loss of cell viability, which enables downstream molecular characterization with scRNA-seq (Methods). **b** Plot of single-cell MAR versus mass for BT159 GBM cells treated with either DMSO (blue circles, *n* = 83) or RG7388 (an MDM2 inhibitor, red triangles, *n* = 66) for 16 h. Kernel density plots, using the same color scheme, are included in the margins for both populations. ***P < 0.001, Mann-Whitney *U* test. **c** Volcano plot showing log-transformed average expression fold change and log-transformed *P*-values (Bonferroni corrected) for genes upregulated (red) or downregulated (blue) in BT159 cells treated with RG7388 as compared with DMSO treatment. **d** Plot of mitosis scores versus buoyant mass for BT159 cells treated with DMSO (blue circles, *n* = 83) or RG7388 (red triangles, *n* = 66) for 16 h. Mitosis scores were calculated by taking the average *z*-score adjusted gene expression values of a panel of mitosis-related genes (*n* = 29, Additional file 10: Table S9; Methods). Kernel density plots, using the same color scheme, are included in the margins for both populations. ***P < 0.001, Mann-Whitney *U* test. **e** Plot of significantly enriched canonical pathways (FDR < 0.05) in RG7388-treated BT159 cells (*n* = 66), as determined by ingenuity pathway analysis, among genes with significant positive (black) or negative (gray) correlations with normalized MAR. (Additional file 1: Figure S4, Additional file 11: Table S10, Methods)

Cancer cell drug responses are known to be highly heterogeneous at the single-cell level [18, 26], and it is now well established that the presence of even a small fraction of cells that are unresponsive to therapy can lead to resistance and recurrence of cancers [34]. Single-cell transcriptional profiling has been shown to provide a powerful means of characterizing such heterogeneity in clinically relevant tissue samples [35, 36], yet the direct interrogation of drug response is still most commonly measured in clinical trials and the laboratory using bulk viability assays [37]. Although effective in quantifying the relative fraction of resistant cells within a heterogeneous population, these assays rely on endpoint measurements. Taken too late, they may miss responding cells (which are lost to cell death) and/or the preceding molecular events that impact survival; taken too early, bulk measurements can muddle the features of responding and non-responding cell subsets (Fig. 4a). However, we have previously shown that, prior to viability loss, single-cell biophysical changes of mass and MAR collected with the SMR can predict response to drug treatment [18]. Therefore, we reasoned that downstream molecular characterization could be used to further contextualize single-cell mass and growth rate heterogeneity both at baseline and in response to perturbation with drug treatment.

To demonstrate a framework for the characterization of single-cell biophysical heterogeneity in the presence or absence of drug, we decided to measure the effect of an MDM2 inhibitor (RG7388, Roche) on BT159 cells, a patient-derived cell line (PDCL) generated from a primary glioblastoma (GBM) (Methods). GBM PDCLs are known to be particularly heterogeneous with respect to cell lineage and have a cancer stem cell like hierarchy proposed to contribute to profound treatment resistance of these tumors [38]. MDM2, meanwhile, typically binds to p53 inhibiting its transcriptional activity and leading to proteasome-mediated degradation [39]. In prior work, we showed pharmacologic inhibition of MDM2 was a promising therapeutic avenue in GBM patients with wild-type TP53 because in preclinical patient derived models, the drug leads to increased expression and stability of p53, significant responses and even tumor regression via induction of apoptotic cell death [40]. However, in vivo testing revealed that, upon withdrawal of MDM2 inhibition, tumors consistently relapsed, suggesting variable response to treatment [40].

To characterize biophysical heterogeneity at the single-cell level, we collected linked mass, MAR and gene expression measurements for single BT159 cells that had either been treated for 16 h with RG7388 or DMSO (control) (Methods). Overall, the drug-treated population of cells showed a marked reduction in average MAR and an increase in average mass as compared to the control population of cells, as expected from cell cycle exit and apoptosis (*P* < 0.001, Mann-Whitney *U* test, Fig. 4b). However, there was also considerable heterogeneity in biophysical response to drug, with some cells continuing to show a positive MAR at the time of measurement (Additional file 1: Figure S8). Since these measurements were collected at a single time point, it is difficult to assess whether the cells that continue to grow in the presence of drug are, in fact, resistant to therapy or simply display a delayed response to treatment. Nonetheless, the biophysical heterogeneity found in these results affords the opportunity to determine transcriptional signatures that correlate with this variability at this particular time point.

We next considered only the transcriptional data. As expected, an unbiased analysis (dimensionality reduction by principal components analysis (PCA) and visualization using a t-stochastic neighbor-embedding (tSNE) plot, Methods) revealed distinct transcriptional profiles for drug-treated and control cell populations (Additional file 1: Figure S9a). Relative to DMSO-treated cells, drug-treated cells displayed gene expression signatures consistent with the mechanism of MDM2 inhibition, with genes positively regulated by p53, such as *CDK1NA* (p21) and *MDM2*, showing significant upregulation, and genes negatively regulated by p53, such as *CDK1* and *CDC20*, showing significant downregulation (Bonferroni-corrected *P* < 0.05, Fig. 4c, Additional file 9: Table S8) [41]. We then performed dimensionality reduction (PCA) and graph-based clustering (*k*-nearest neighbors, KNN) on the transcriptional data from the drug-treated cells alone and visualized our results using a tSNE plot (Additional file 1: Figure S9b; Methods). This clustering analysis did not reveal any clear subsets of drug-treated cells with distinctly different responses to MDM2 inhibition.

Since our transcriptional measurements suggested that all MDM2-inhibitor treated cells were actively experiencing drug but our biophysical measurements revealed mass and MAR heterogeneity, we decided to explicitly examine whether the linked nature of our measurements could be used to shed light on the drivers of biophysical variability at this time point after treatment with DMSO or RG7388. When examining linked measurements of gene expression and cell mass in DMSO treated cells, we found that genes ranked by correlation strength with mass were highly enriched for functional annotations relating to cell cycle progression (Additional file 2: Tables S1, Additional file 3: Table S2). Also, as with the other cell types presented here, larger cells in the control population expressed a higher level of genes associated with late cell cycle events, specifically mitosis (Fig. 4d, Additional file 10: Table S9). Interestingly, an unsupervised clustering analysis (PCA followed by KNN clustering, Methods) of the DMSO-treated cells alone revealed two distinct subsets which had significantly different average masses (*P* < 0.01, Mann-Whitney *U* test, Additional file 1: Figure S9c,d), and an upregulation of genes relating to cell cycle progression in the subset with a larger average mass (Additional file 1: Figure S9e).

MDM2 inhibitor-treated cells, meanwhile, showed significantly reduced expression of mitosis-specific genes (*P* < 0.001, Mann-Whitney *U* test, Fig. 4d). Moreover, in these cells, we did not observe any significant cell cycle-related functional enrichments among those genes correlated with cell mass (FDR > 0.05). These results demonstrate that upon MDM2 inhibition and stabilization of p53 signaling in these cells, cell cycle arrest is achieved as expected but there is no longer a correlation between cell mass and cell cycle-related gene expression (*ρ* = 0.47, *P* < 0.001 for DMSO-treated cells; *ρ* = − 0.07, *P* = 0.54 for drug-treated cells). Furthermore, since a subset of cells within the drug-treated population displayed a positive MAR despite ablated cell cycle gene expression (Fig. 4b), our data suggest that cell cycle gene expression alone does not fully account for variability in the single-cell biophysical response. In fact, we did not observe a significant correlation between PCs computed for the drug-treated single-cell transcriptomes and any biophysical properties measured (*P* > 0.05; Methods).

To determine transcriptional signatures that may underlie this biophysical heterogeneity, we utilized the corresponding single-cell MAR data to further contextualize gene expression. Genes ranked by correlation strength with mass-normalized MAR in the MDM2 inhibitor-treated population of cells showed a significant negative enrichment (i.e., higher expression in cells accumulating less mass over time) for functional annotations related to apoptosis regulation, specifically related to p53 signaling (FDR < 0.05, Additional file 2: Table S1, Additional file 3: Table S2). The DMSO-treated population of cells, meanwhile, did not show any significant functional enrichments among genes ranked by correlation with normalized-MAR (FDR > 0.05, Additional file 2: Table S1, Additional file 3: Table S2). Similarly, ingenuity pathway analysis (IPA, Qiagen) performed on drug-treated cells revealed significant enrichment of canonical apoptosis signaling among genes showing significant negative correlations with normalized MAR (FDR < 0.05, Fig. 4e) while the same analysis on DMSO-treated cells did not reveal any apoptosis-related signaling significantly correlated with MAR (FDR > 0.05, Additional file 1: Figure S10, Additional file 11: S10). Together, these results suggest that cells with a higher normalized MAR had a lower expression of genes related to apoptotic signaling orchestrated by p53, but only in drug treated cells, consistent with the mechanisms of MDM2. IPA of drug-treated cells further revealed partial enrichment (FDR = 0.09) for PTEN signaling (a negative regulator of AKT) and significant enrichment (FDR < 0.05) for mTOR signaling (a positive regulator of AKT) among genes significantly negatively and positively correlated with normalized MAR, respectively [42, 43]. IPA of DMSO treated cells, however, did not reveal significant enrichment for mTOR or PTEN signaling (FDR > 0.1) in genes correlated with normalized MAR (Additional file 1: Figure S10, Additional file 11: Table S10). Together, these results suggest that cells which continue to grow in the presence of MDM2 inhibition may exhibit more stable AKT signaling, which itself drives MDM2 expression, as compared with cells with decreased normalized MAR, pointing to a potential mechanism of cell survival in the presence of treatment [44, 45]. Though preliminary, these results demonstrate the unique insights offered by linked measurements of biophysical phenotype and gene expression when examining cancer cell drug response at the single-cell level.

## Conclusion

The platform presented here enables linked measurements of single-cell biophysical properties and gene expression. Having demonstrated the resolution and reproducibility of these linked data sets with measurements of stable cell lines (L1210 and FL5.12 cells), we present frameworks for two key applications of these linked data sets (i) characterizing immune cell activation and differentiation and (ii) examining cancer cell drug response at the single-cell level.

While the primary focus of this work was on conducting scRNA-seq downstream of the sSMR, we also envision this platform being a useful tool for linking biophysical data with other recently developed approaches that enable DNA sequencing, epigenomic characterization, or multi-omic measurements of single cells [6, 7, 46].

We believe that these linked measurements will offer a novel means of exploring a range of biological questions. For instance, when paired with recently developed computational approaches, these linked biophysical and transcriptional measurements may offer insights into cell cycle regulation as well as provide an additional approach for addressing the potentially confounding effects of cell cycle in scRNA-seq analyses [23]. Clinically, mass and MAR have proven to be effective biomarkers for characterizing cancer cell drug susceptibility at the single-cell level [18, 26]. The ability to link these biophysical measurements with gene expression or genetic profiling offers the exciting opportunity to move beyond the simple classification of responding and non-responding cells and to begin to explore the molecular mechanisms that may drive such behaviors. We envision that this and related approaches may one day inform more effective precision medicine pipelines [47].

## Methods

### Cell culture and primary cell preparation

L1210 murine lymphocytic leukemia cells (ECACC) were cultured in RPMI 1640 (Gibco) with 10% fetal bovine serum and 1% antibiotic-antimycotic (Gibco). FL5.12 murine pre-B cells (gift from the Vander Heiden Lab, MIT) were cultured in the same media with the addition of 10 ng/ml IL-3 (R&D Systems). For all growth and collection experiments, cells were passaged to a concentration of 5 × 10^5^ cells/ml the night before to ensure consistent culture confluence at time of measurement.

Naïve CD8+ T cells were isolated from a 13 week old, male, C57BL/6 J mouse. Splenocytes were subject to red blood cell lysis with ACK buffer (Gibco) followed by naïve CD8+ T cell isolation using a MACS-based isolation kit (Miltenyi Biotec). Purified cells were cultured in RPMI 1640 (Gibco) with 10% fetal bovine serum, 55 μM 2-mercaptoethanol (Gibco), 1% antibiotic-antimycotic (Gibco) and 100 U/ml IL2 (Peprotech). The naïve CD8+ T cells were activated in vitro with 5 μg/ml plate-bound anti-mouse CD3 (clone: 145-2c11, BioLegend), 0.5 μg/ml plate-bound ICAM-1/CD54 (R&D Systems), and 2 μg/ml soluble anti-mouse CD28 (clone: 37.51, BioLegend). Cells were seeded at a concentration of 1 × 10^6^ cells/ml in a 96 well plate and activated for either 24 or 48 h prior to measurement in the sSMR.

Primary GBM cells used to generate the BT159 line were harvested from excess tissue resection specimens through cycles of enzymatic (neural tissue dissociation kit with papain, Miltenyi Biotec) and mechanical dissociation in a tissue grinder (gentleMACS dissociator, Miltenyi Biotec). Cells were grown as tumorspheres in NeuroCult NS-A proliferation media (Miltenyi Biotec) supplemented with 2 μg/ml Heparin, 20 ng/ml human epidermal growth factor (EGF), 10 ng/ml human bFGF in ultra-low attachment coated flasks (Corning). Prior to measurement, the BT159 cells were dissociated with Accutase (Sigma-Aldrich) at 37 °C for 7 min. For drug experiments, cells were treated with 250 nM of the MDM2 inhibitor RG7388 (Roche) or DMSO for 16 h prior to dissociation for measurement.

### Single-cell growth measurements and collection

For all experiments, cells were adjusted to a final concentration of 2.5 × 10^5^ cells/ml to load single cells into the mass sensor array as described in Additional file 1: Note S1. Single-cell growth measurements were conducted as described previously [17]. In order to exchange buffer and flush individual cells from the system, the release side of the device was constantly flushed with PBS at a rate of 15 μL per minute (Additional file 1: Figure S1, P2 to P4). Upon detection of a single-cell at the final cantilever of the sSMR, as indicated by a supra-threshold shift in resonant frequency, a set of three-dimensional motorized stages (ThorLabs) was triggered to move a custom PCR-tube strip mount from a waste collection position to a sample collection position. The location of these motors was written to a file for the duration of the experiment in order to annotate single-cell mass and MAR measurements with well position, and thus transcriptional profiles, downstream. Each cell was collected in 5 μl of PBS directly in to a PCR tube containing 5 μl of 2× TCL lysis buffer (Qiagen) with 2% *v*/*v* 2-mercaptoethanol (Sigma) for a total final reaction volume of 10 μl. After each 8-tube PCR strip was filled with cells, the strip was spun down at 1000 g for 30 s and placed immediately on dry ice. Following collection, samples were stored at − 80 C prior to library preparation and sequencing.

### scRNA-seq

Single-cell RNA isolation, cDNA library synthesis, next generation sequencing, read alignment and gene expression estimation were performed as described previously [48]. Briefly, Smart-Seq2 whole transcriptome amplification and library preparation were performed on single-cell lysates collected with the sSMR [49]. Single-cell libraries were then sequenced on an Illumina NextSeq 500 using 30-bp paired end reads. Data was initially filtered to exclude cell doublets or cells with failed matching of masses for growth rate measurement. This step left 87 out of 96 total L1210 cells, 144 out of 192 total FL5.12 cells, 178 out of 192 total CD8+ T cells, and 181 out of 192 total BT159 GBM cells. Next, cells that exceeded a preliminary complexity threshold (4000 genes for L1210 and FL5.12 cells, 2000 genes for CD8+ T cells, or 1000 genes for BT159 cells) were selected for further analysis. Overall, this yielded 85 out of 87 total L1210 cells, 124 out of 144 total FL5.12 cells, 108 out of 178 total CD8+ T cells, and 149 out of 192 total BT159 cells. These cells selected for analysis were sequenced to an average depth of 1,698,879 + 106,027 (s.e.m.) reads for L1210 cells, 760,919 + 36,679 (s.e.m.) reads for FL5.12 cells, 1,333,686 + 90,744 (s.e.m.) reads for CD8+ T cells, and 993,629 + 75,796 (s.e.m.) reads for BT159 cells respectively. Reads were aligned using TopHat2 and expression estimates (transcripts per million; TPM) for all UCSC-annotated mouse genes (mm10, for L1210, FL5.12, and CD8+ T cells) or human genes (hg19, for BT159 cells) were calculated using RNA-seq by expectation maximization (RSEM) [50, 51]. The average transcriptome alignments were 67.4 + 0.38% (s.e.m.) for L1210 cells, 64.8+ 0.51% (s.e.m.) for FL5.12 cells, 57.3 + 1.36% (s.e.m.) for CD8+ T cells, and 35.2 + 0.84% (s.e.m.) for BT159 cells. The average number of genes detected was 7,207 + 94 (s.e.m.) for L1210 cells, 6,891 + 81 (s.e.m.) for FL5.12 cells, 5,149 + 159 (s.e.m.) for CD8+ T cells, and 5,347 + 173 (s.e.m.) for BT159 cells (Additional file 1: Figure S2).

### Gene expression analysis

All analysis was performed on log-transformed expression level measurements (ln(TPM + 1)). Data pre-processing was conducted with the Seurat package for R [10]. All genes that were detected in > 5% of cells were included in the final analysis for each group of cells (L1210, FL5.12, CD8+ T cells, and BT159 GBM cells).

#### Significance-testing

To define the null distribution of correlation coefficients described in Fig. 3, we determined the Spearman correlation between cell cycle gene expression levels and mass for randomly shuffled data sampled from the experimental values (i.e., mismatching single-cell mass and gene expression data). After 10,000 iterations, we used the average mean and standard deviation values of these correlation coefficient distributions to define the null distributions presented.

We computed the null distributions for the correlation coefficients between either mass, MAR, or normalized MAR and the principal components for either the DMSO-treated, drug-treated, or combined transcriptomic data sets using a similar random shuffling of PC coordinates across single-cells. Following 10,000 iterations, the mean and standard deviation of these distributions were compared to the correlation of each biophysical parameter with all significant principal components (PCs). For each data set, the PCElbow plot and jackstraw functions in Seurat were used to select significant PCs whose explained variation preceded a precipitous drop in cumulative explained variation (elbow). In each data set, for consistency, the top 10 PCs were investigated, although in some cases fewer than 10 PCs preceded the elbow. Correlation coefficients were deemed insignificant if they were within two standard deviations of the mean determined from random shuffling.

#### Gene set enrichment analysis

Ranked gene lists were created for each cell population by determining the gene-wise correlation coefficient (Spearman) between log-transformed gene expression levels and either single-cell mass or growth efficiency (MAR/mass; Additional file 2: Table S1). Spearman and Pearson correlation coefficients yielded similar results for all conditions measured (Additional file 1: Figure S4). Gene set enrichment was computed for these ranked lists using the GSEA Preranked tool, implemented with the fgsea package in R (Additional file 3: Table S2) [21, 52].

#### Differential expression

Differential expression analysis for the 24 versus 48 h CD8+ T cell measurements, as well as the DMSO versus RG7388 treated BT159 cells, was performed using the FindMarkers function of Seurat with the Wilcoxon rank sum test (Additional file 6: Table S5, Additional file 9: Table S8). For the CD8+ T cells, genes were also ranked by log-normalized fold-change expression difference between the 24 and 48 h time points and analyzed with the GSEA Preranked tool (Additional file 7: Table S6, Additional file 6: Table S5). All *P* values presented are Bonferroni corrected, as per Seurat documentation recommendation.

#### Dimensionality reduction

Variable genes for the DMSO-treated, drug-treated, and combined data sets were identified using Seurat’s FindVaribleGenes. Principal components analysis (PCA) was performed over these genes for each of the three sets of cells, followed by non-linear dimensionality reduction by t-stochastic neighbor embedding (tSNE). Clusters were identified in the linear PC space using *K*-nearest neighbor (KNN) clustering, and cluster assignments were visualized on the non-linear tSNE space. For the DMSO-treated cells, we detected two distinct clusters (Additional file 1: Figure S9c); for the RG7388 treated cells, we only detected one (Additional file 1: Figure S9b).

#### Ingenuity pathway analysis

Ingenuity pathway analysis (IPA, Qiagen) was performed on canonical pathways using genes which significantly correlated positively and negatively with normalized MAR (Additional file 1: Figure S4). Briefly, correlation and *P* values for significant genes were uploaded into IPA and analyzed using the “Core Analysis” function. Correlations were input as “Expression: Other” measurements with range from -INF to INF. Significant canonical pathways and upstream regulators (determined by hypergeometric test) with positive and negative *z*-scores are plotted in Fig. 4e.

## Additional files

Additional file 1: Supplementary figures and notes. (PDF 14878 kb)

Additional file 2: **Table S1.** Gene lists ranked by correlation with either mass or mass-normalized MAR for L1210, FL5.12, CD8+ T cells (24 and 48 h activations), and BT159 GBM cells (DMSO and RG7388 treated) with corresponding Spearman correlation coefficients. Genes that are either significantly positively or negatively correlated with the biophysical measurement of interest (as described in Additional file 1: Figure S4) are highlighted in red. (XLSX 3597 kb)

Additional file 3: **Table S2.** Gene set enrichment reports for all the ranked gene lists presented in Additional file 2: Table S1. Enrichments were generated using the fgsea tool in R. Only gene sets with a false discovery rate (FDR) value less than 0.1 are included. (XLSX 88 kb)

Additional file 4: **Table S3.** Cell cycle genes significantly correlated with cell mass for L1210 and FL5.12. Genes from the “chromosome segregation” gene ontology term that had a significant positive correlation with cell mass (*n* = 58 and 31 genes for L1210 and FL5.12 cells, respectively) and genes from the “DNA replication” gene ontology term with a significant negative correlation with cell mass (*n* = 11 and 8 genes for L1210 and FL5.12 cells, respectively) were used to construct the lists for each cell type. Significance was determined as described in Additional file 1: Figure S4. (XLSX 9 kb)

Additional file 5: **Table S4.** List of G1S related genes correlating with normalized growth rate in FL5.12 cells. Genes from the “cell cycle G1 S phase transition” gene ontology term that showed a significant positive correlation with normalized growth rate in FL5.12 cells (*n* = 13 genes, as described in Additional file 1: Figure S7) were used to construct this gene list. (XLSX 8 kb)

Additional file 6: **Table S5.** List of significantly differentially expressed genes between the 24 and 48 h time points for the activated CD8+ T cells with corresponding Bonferroni-corrected *P* values and log-normalized fold change values. Negative values indicate genes expressed at a higher level in the 48 h time point. (XLSX 24 kb)

Additional file 7: **Table S6.** CD8+ T cell gene list ranked by log-normalized fold change in gene expression between the 24 and 48 h activation time points. Negative values indicate genes expressed at a higher level in the 48 h time point. (XLSX 43 kb)

Additional file 8: **Table S7.** Gene set enrichment report for the ranked gene list presented in Additional file 7: Table S6. Enrichments were generated using the fgsea tool in R. Only gene sets with a false discovery rate (FDR) value less than 0.1 are included. (XLSX 17 kb)

Additional file 9: **Table S8.** List of significantly differentially expressed genes between the DMSO and RG7388 treated BT159 GBM cells with corresponding Bonferroni-corrected P values and log-normalized fold change values. Negative values indicate genes that were expressed at a higher level in the DMSO treated cells. (XLSX 451 kb)

Additional file 10: **Table S9.** List of mitosis related genes correlating with mass in DMSO treated BT159 GBM cells. Genes from the “mitosis” gene ontology term that showed a significant positive correlation with cell mass in the DMSO treated BT159 GBM cells (*n* = 29 genes, as described in Additional file 1: Figure S4) were used to construct this gene list. (XLSX 8 kb)

Additional file 11: **Table S10.** Table of ingenuity pathway analysis (IPA) results for canonical pathway analysis of genes significantly positively or negatively correlated with normalized MAR in RG7388 or DMSO treated BT159 cells (Additional file 1: Figure S4, Fig. 4, Additional file 1: Figure S10, Methods). The table includes all pathways with an FDR < 0.1. (XLSX 20 kb)

## Abbreviations

FDR: False discovery rate
MAR: Mass accumulation rate
scRNA-seq: Single-cell RNA sequencing
sSMR: Serial suspended microchannel resonator
