# Supplementary material for: Linking single-cell measurements of mass, growth rate, and gene expression
Source: Genome Biol. 2018 Nov 27;19:207. doi: 10.1186/s13059-018-1576-0 (PMC6260722; doi:10.1186/s13059-018-1576-0)
Supplement: Supplementary file 1 — Supplementary figures and notes. (PDF 14878 kb) [file 13059_2018_1576_MOESM1_ESM.pdf]

Figure S1

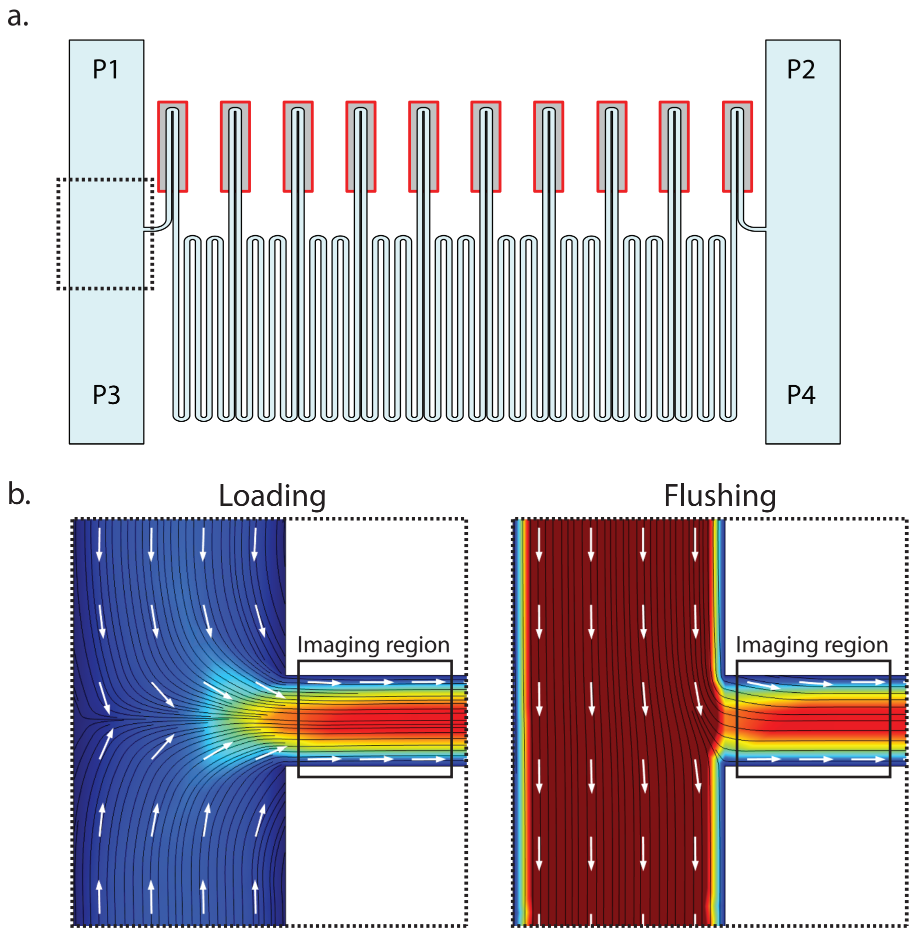

Figure S1 | Fluidic regimes for maintaining cell spacing in the sSMR
(a) Schematic of sSMR presented in **Figure 1** denoting the array entrance region used for fluidic simulation presented in **(b)** (dashed box outline). **(b)** COMSOL fluidic simulations demonstrating the loading (left) and flushing (right) fluidic regimes described in **Additional File 1: Note S1**. The imaging region used to trigger between each fluidic state is outlined (solid box).

Figure S2

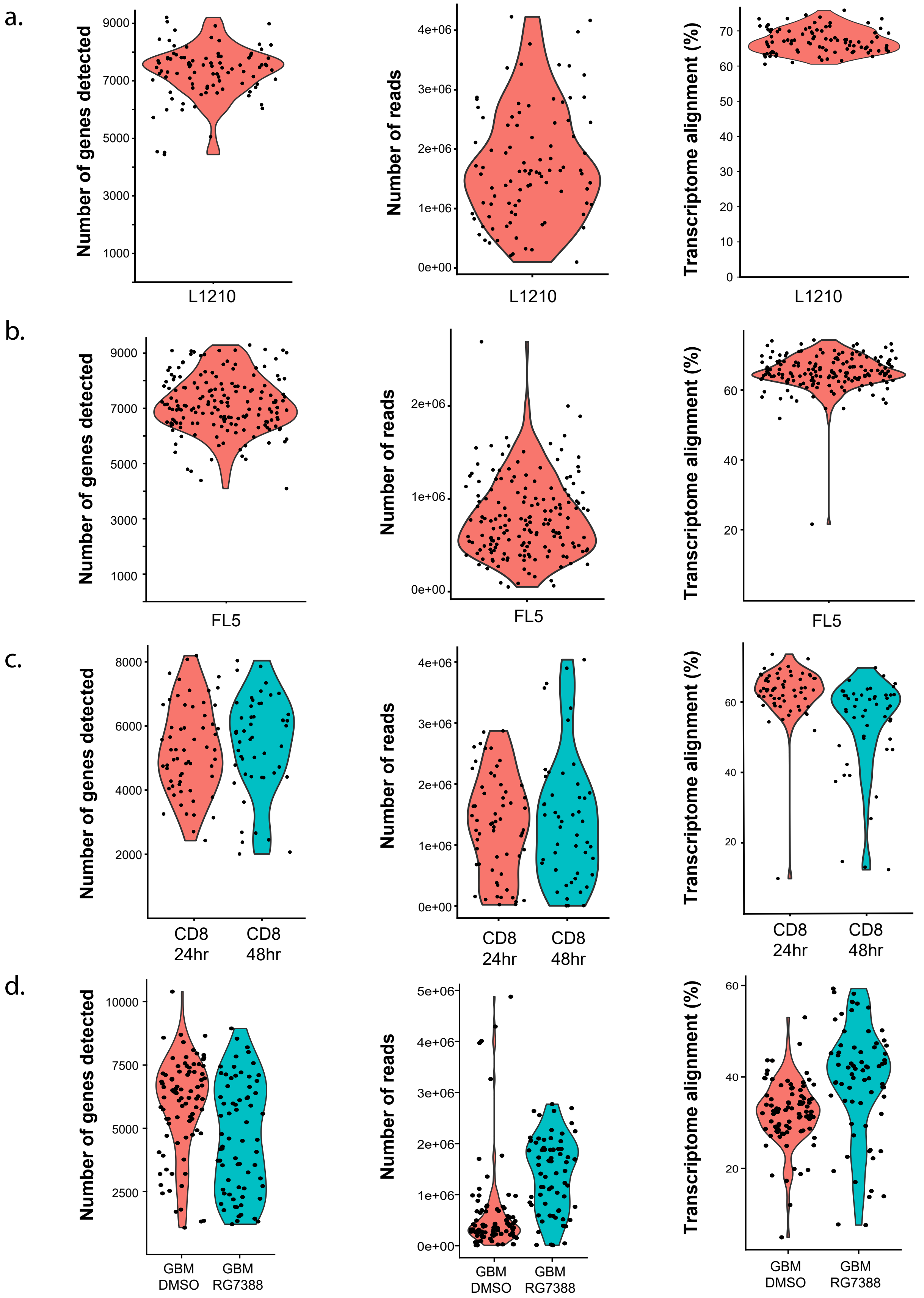

Figure S2 | Quality metrics for scRNA-seq libraries

Violin plots and overlaid points showing the number of genes detected (left), sequencing depth (center), and transcriptome alignment (right) for each scRNA-seq library prepared for **(a)** L1210 cells, **(b)** FL5.12 cells **(c)** CD8+ T cells activated for either 24 or 48h (red and blue outlines, respectively), and **(d)** BT159 GBM cells treated with either DMSO or RG7388 (red and blue outlines, respectively) that passed initial quality thresholds and were used for further analysis (**Methods**).

Figure S3

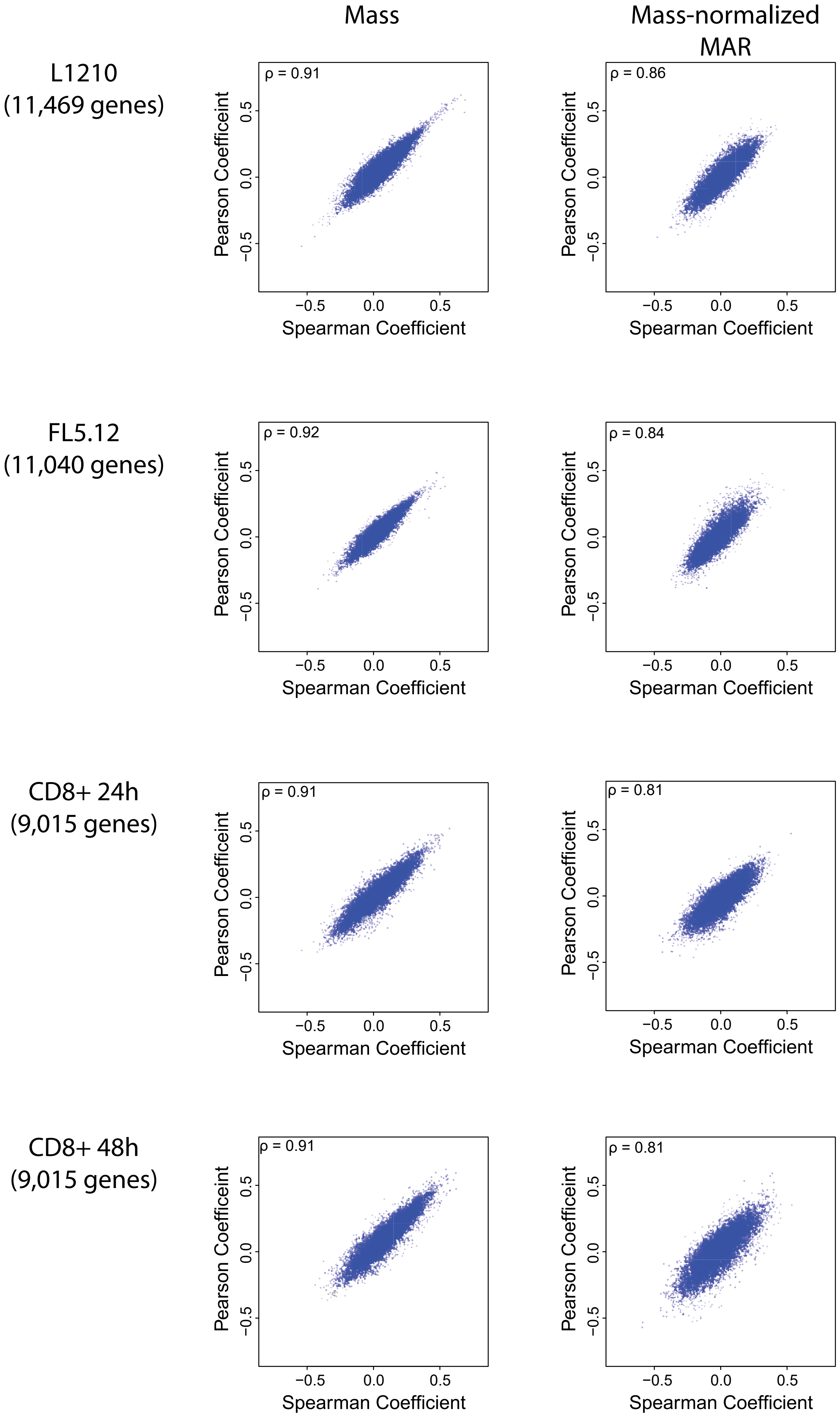

Mass
Mass-normalized MAR
L1210
(11,469 genes)
ρ = 0.91
ρ = 0.86
FL5.12
(11,040 genes)
ρ = 0.92
ρ = 0.84
CD8+ 24h
(9,015 genes)
ρ = 0.91
ρ = 0.81
CD8+ 48h
(9,015 genes)
ρ = 0.91
ρ = 0.81
Pearson Coefficeint
Spearman Coefficient
−0.5
0.0
0.5

Figure S3 (continued)

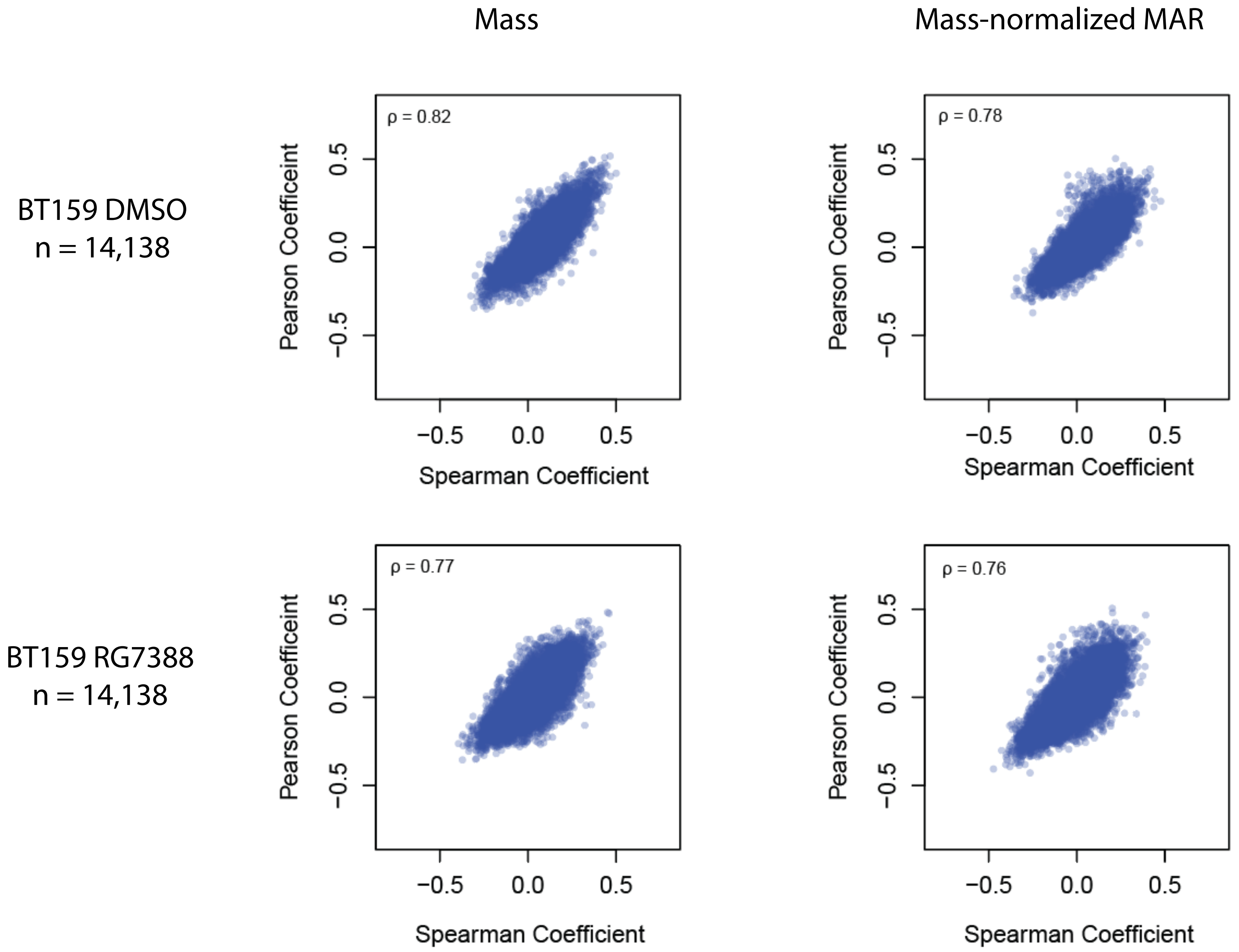

Figure S3 | Comparison of Pearson and Spearman coefficients for correlations between gene expression and biophysical parameters

Plots of the Pearson coefficient versus Spearman coefficient for expression level correlations with either mass (left column) or mass-normalized MAR (right column) for L1210, FL5.12, CD8+ T cells (24 and 48h time points), and BT159 GBM cells (DMSO and RG7388 treated). Each cell type lists the total number of genes being compared and each plot indicates the Spearman coefficient between the Spearman and Pearson coefficients across all genes. Each measurement set reveals similar gene-level rankings for both Spearman and Pearson coefficients.

Figure S4

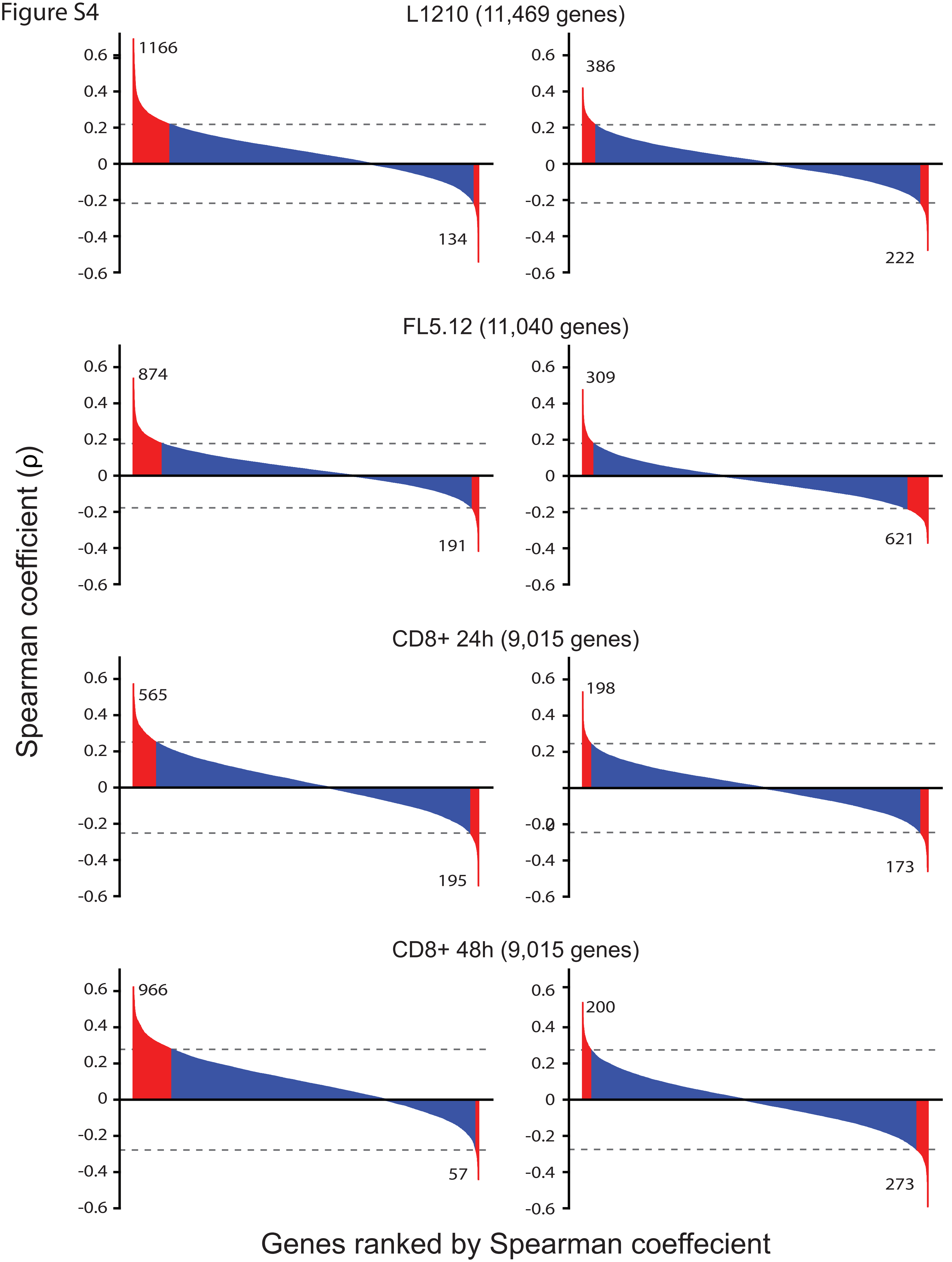
L1210 (11,469 genes)
1166
134
386
222
FL5.12 (11,040 genes)
874
191
309
621
CD8+ 24h (9,015 genes)
565
195
198
173
CD8+ 48h (9,015 genes)
966
57
200
273
Spearman coefficient (ρ)
0.6
0.4
0.2
0
-0.2
-0.4
-0.6
Genes ranked by Spearman coeffecient

Figure S4 (continued)

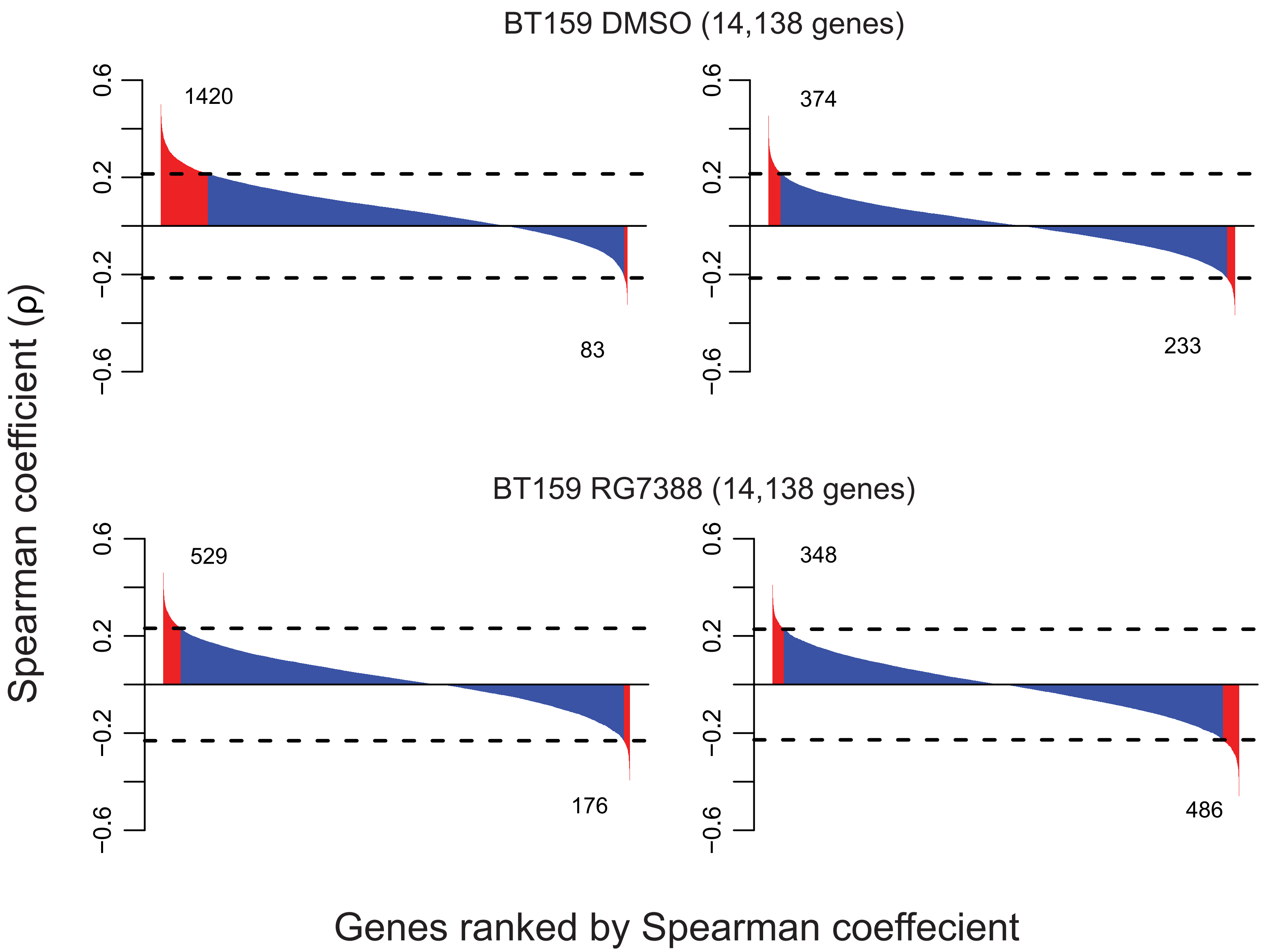

Figure S4 | Expression level correlation with biophysical parameters for L1210, FL5.12, CD8+ T cells, and BT159 GBM cells

Bar plots denoting the correlation strength of individual gene's expression levels with either mass (left) or mass-normalized MAR (right) for (a) L1210 cells (n = 11,469 genes), (b) FL5.12 cells (n = 11,040 genes), (c) CD8+ T cells after 24h of activation (n = 9,015 genes), (d) CD8+ T cells after 48h of activations (n = 9,015 genes), (e) BT159 cells treated with DMSO (n = 14,138 genes), and (f) BT159 cells treated with RG7388 (n = 14,138 genes). Genes are plotted in rank order where genes with highest positive and negative correlations with biophysical parameters are found at the left-most and right-most portion of the x axis, respectively. For each data set, a null distribution of correlation coefficients was determined by finding the correlation between gene expression and mass for randomly permuted data. After 10 iterations, we determined the average standard deviation of these distributions of correlation coefficients. Any individual gene that had a correlation coefficient with an absolute value greater than twice the standard deviation ($P<0.05$, denoted by the dashed lines in the plots) was considered significant (red bars), all genes presented as blue bars fell below this threshold. The number of genes showing a significant positive or negative correlation with the biophysical parameter of interest are shown in each plot.

Figure S5

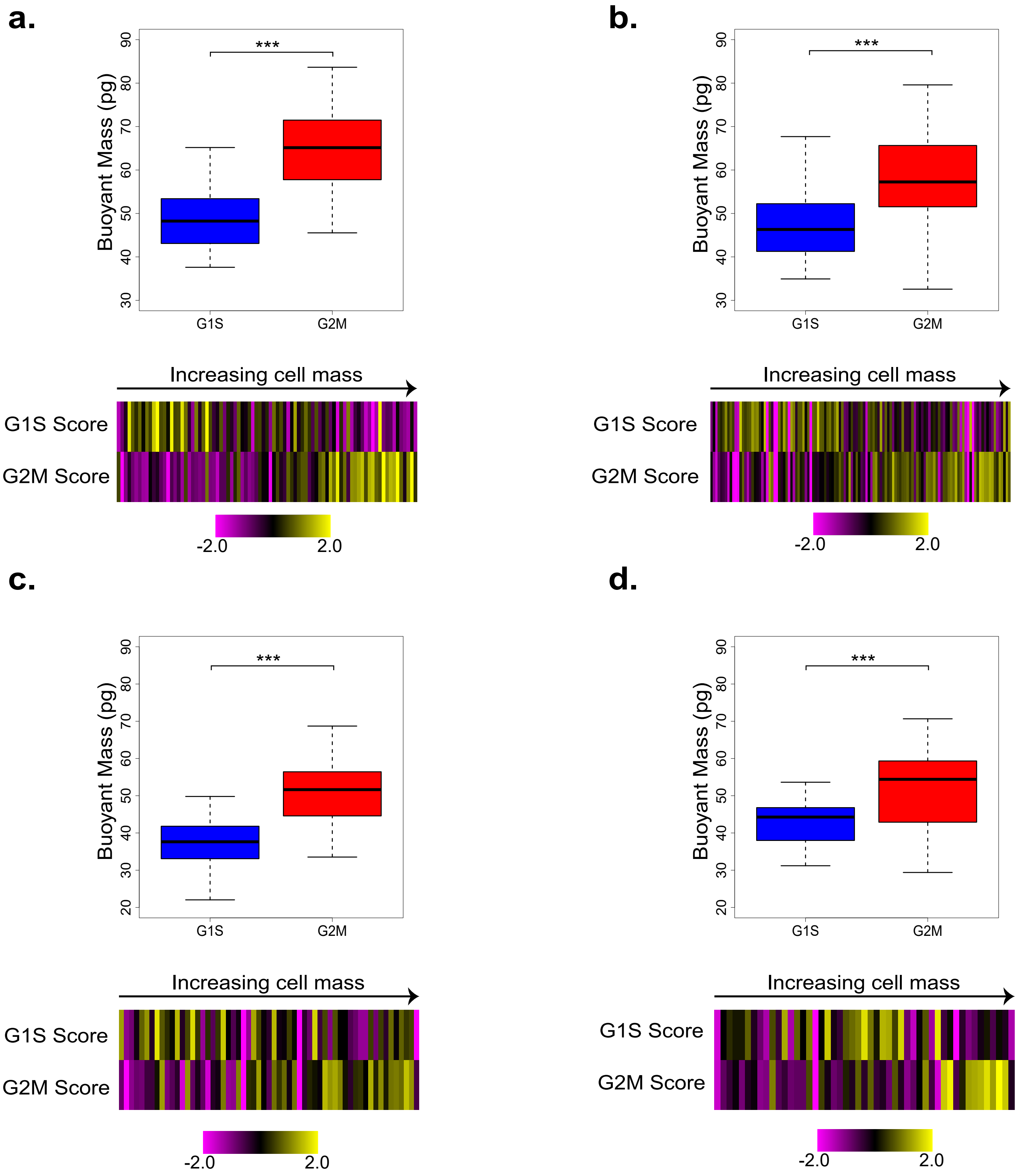

Figure S5 | Comparison of cell cycle gene expression and cell mass

Boxplots (top) showing the mass distribution of single-cells classified as being in the G1/S or G2/M phases of the cell cycle as described in **Additional File 1: Note S3** for **(a)** L1210 cells (n = 48 and 37 for G1/S and G2/M, respectively), **(b)** FL5.12 (n = 63 and 61 for G1/S and G2/M, respectively), **(c)** CD8+ T cells after 24h of activation (n = 25 and 34 for G1/S and G2/M, respectively), and **(d)** CD8+ T cells after 48h of activation (n = 25 and 24 for G1/S and G2/M, respectively) (*** indicates P<0.001, Mann-Whitney U Test). Below each boxplot is a heatmap showing the G1/S and G2/M expression scores for cells ranked by buoyant mass, also as described in **Additional File 1: Note S3**.

Figure S6

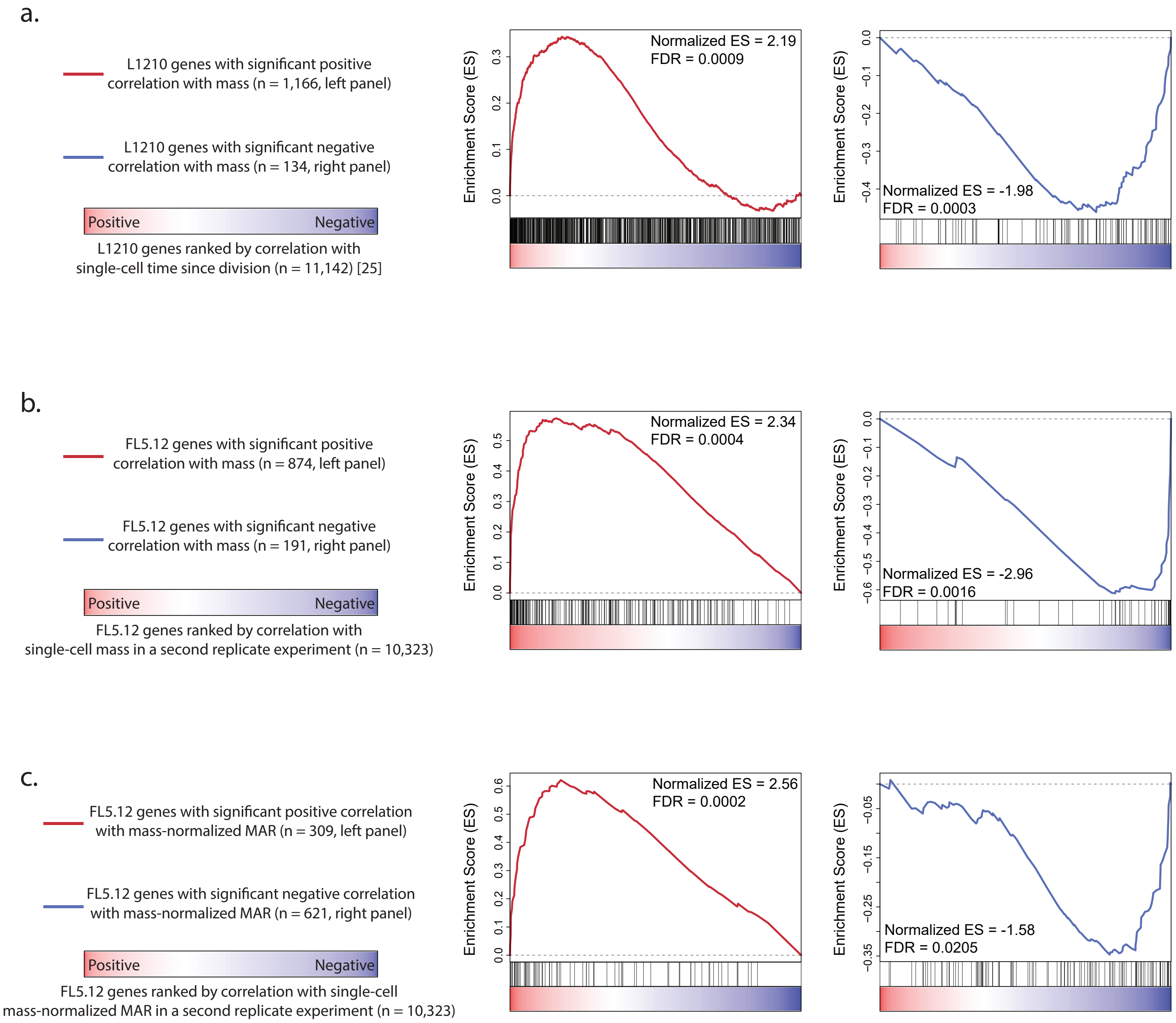

Figure S6 | Reproducibility of linked measurements for L1210 and FL5.12 cells
(a) Enrichment plots for genes with significant positive (left, n = 1,166) or negative (right, n = 134) correlations with single-cell mass amongst genes ranked by expression level correlation with time since division in L1210 cells – determined by Kimmerling et al. (**Additional File 1: Note S2**) [25]. Significant enrichment (FDR = 0.0009 and 0.0003 for positive and negative sets, respectively) suggests that a consistent cell cycle gene expression signature correlates with both cell mass and time since division in L1210 cells. **(b)** Enrichment plots for genes with significant positive (left, n = 874) and negative (right, n = 191) correlations with FL5.12 cell mass amongst a full gene list ranked by expression level correlation with FL5.12 cell mass from a second, independent experiment. The significant enrichment here (FDR = 0.0004 and 0.0016 for positive and negative sets, respectively) demonstrates a reproducible gene expression signature corresponding to FL5.12 mass. **(c)** Same analysis as in **(b)** for genes that correlated significantly with mass-normalized growth rate (growth efficiency, n = 309 and 621 genes for positive and negative correlations, respectively) as opposed to mass, demonstrating reproducible growth-related gene expression signatures as well (FDR = 0.0002 and 0.0205 for positive and negative sets, respectively).

Figure S7

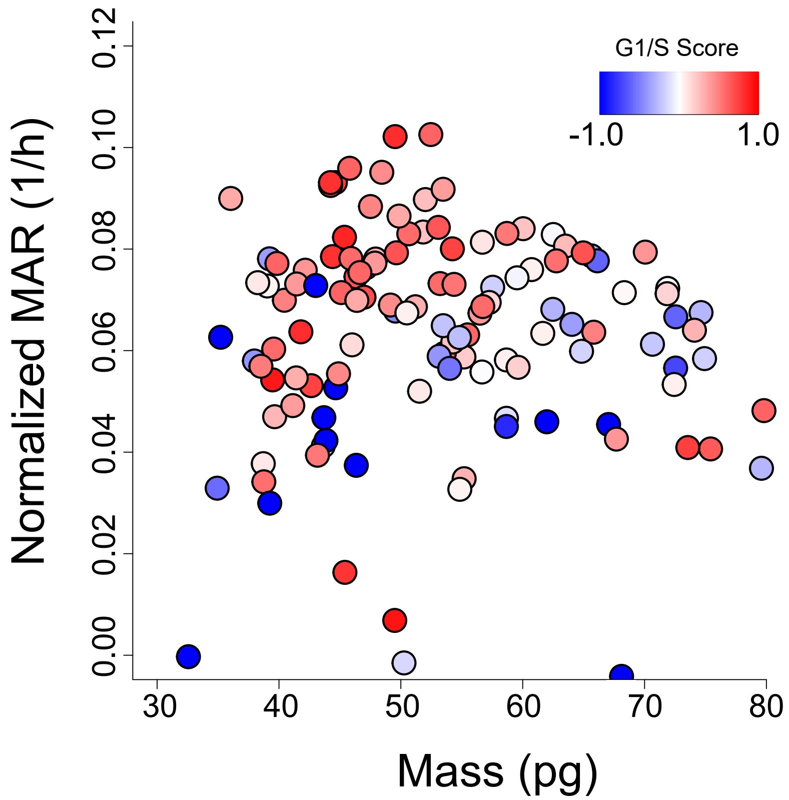

Figure S7 | Cell cycle gene expression versus normalized growth rate in FL5.12 cells

Plot of mass versus mass-normalized growth rate (growth efficiency) for a subset of the FL5.12 cells depicted in **Figure 2** that were captured downstream for scRNA-seq (n = 124). Points are colored by G1/S score. The “cell cycle G1/S phase transition” gene ontology term was found to be significantly enriched amongst genes ranked by correlation with growth efficiency. To determine the G1/S transition scores for single FL5.12 cells, we found the average of mean-centered, z-score scaled expression values for the genes in the “cell cycle G1/S phase transition” gene ontology term found to correlate significantly with normalized growth rate (n = 13 genes, **Additional File 1: Figure S4**, **Additional File 5: Table S4**) [21].

Figure S8

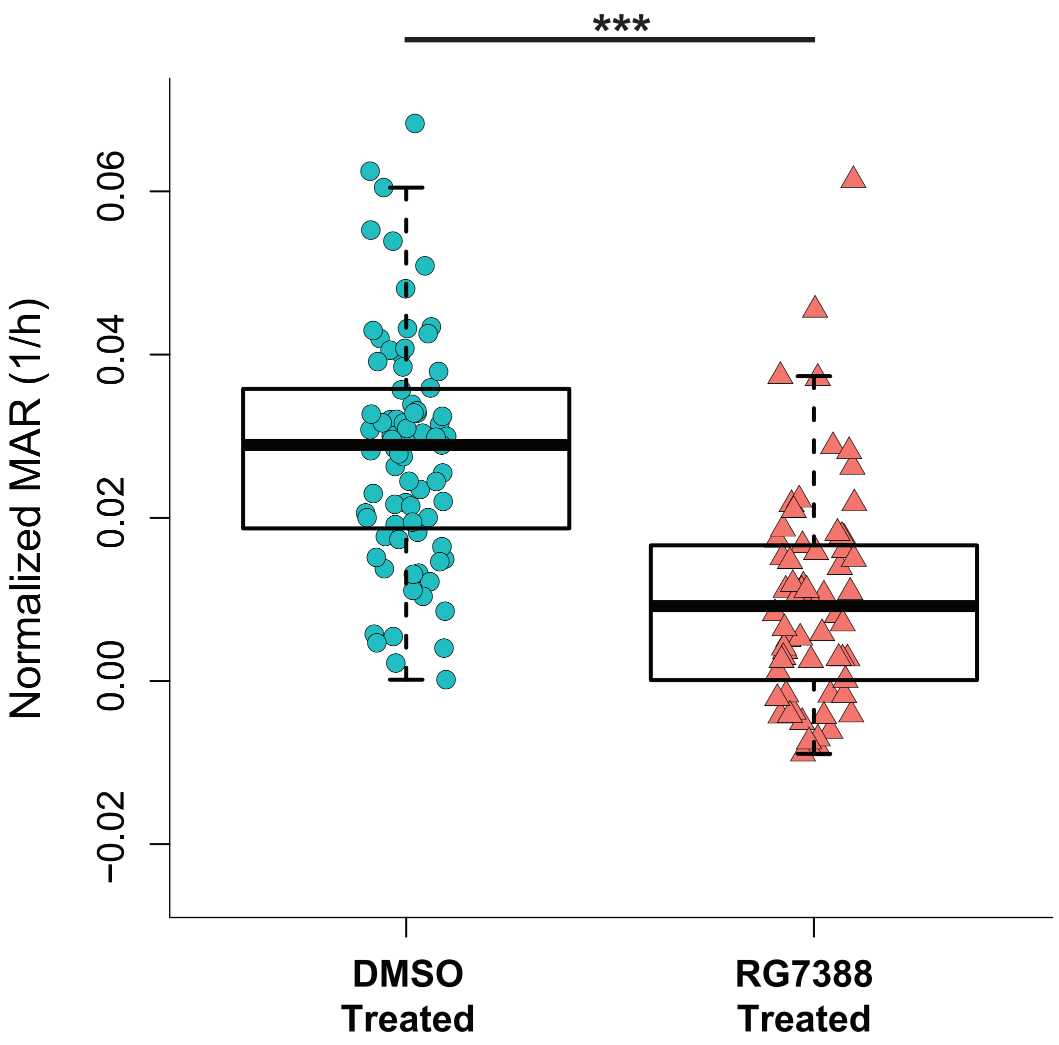

Figure S8 | Mass-normalized MAR measurements for BT159 cells

Single-cell mass-normalized MAR measurements and corresponding boxplots for BT159 cells treated for 16h with either DMSO (left, n= 83) or RG7388 (right, n = 66). *** indicates $P < 0.001$, Mann-Whitney U-test.

Figure S9

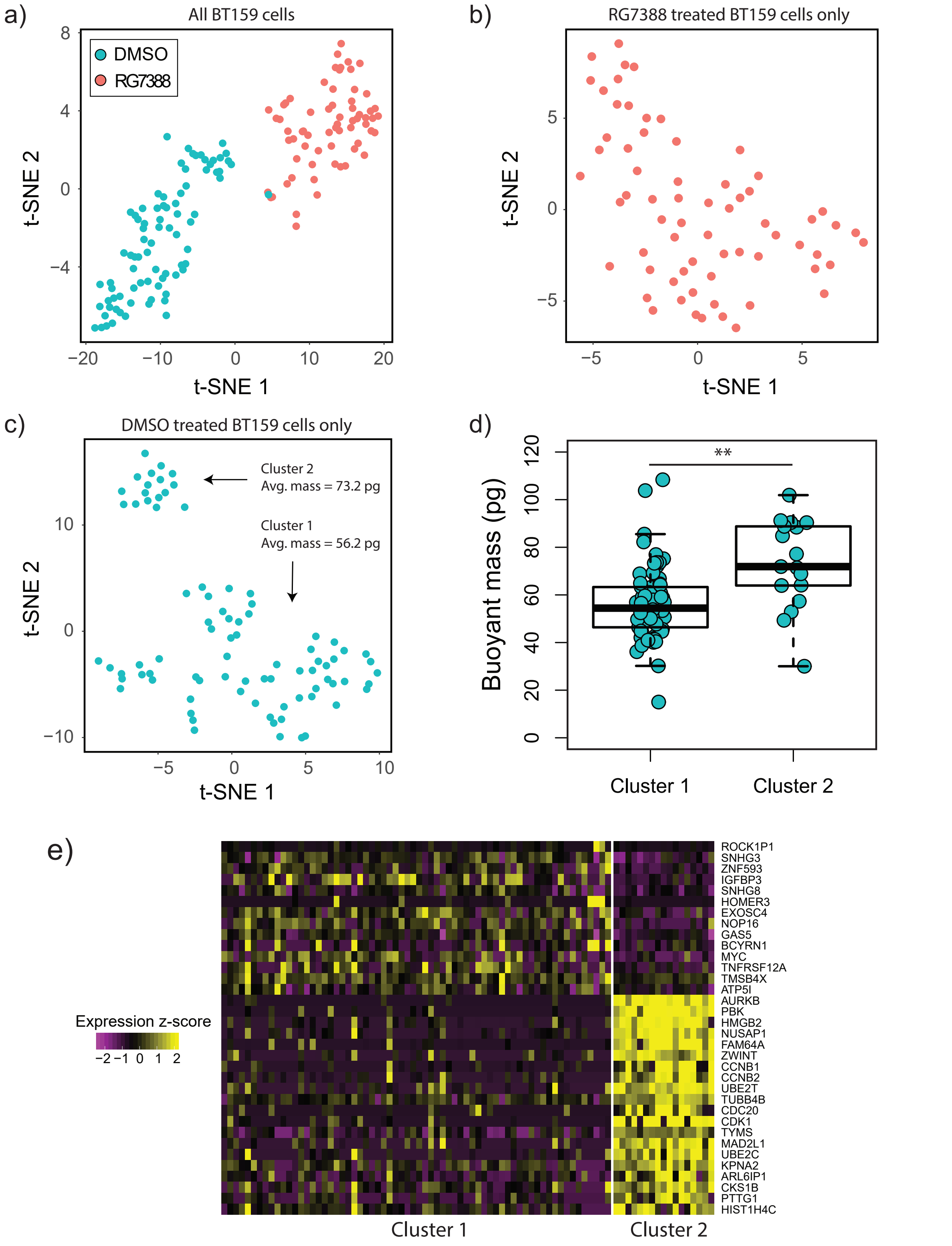

Figure S9 | *t*-SNE analysis of BT159 cells

(a) t-SNE plot of both DMSO treated (n = 83, blue points) and RG7388 treated (n = 66, red points) BT159 cells analyzed together. **(b)** t-SNE plot of RG7388 treated BT159 cells alone. **(c)** t-SNE plot of DMSO treated BT159 cells alone. Annotations indicate the two distinct subpopulations identified by KNN clustering over significant PCs and the corresponding average masses of cells within these clusters. **(d)** Single-cell mass measurements and corresponding boxplots for two clusters (n = 66 and n = 17 for Cluster 1 and Cluster 2, respectively) of DMSO treated BT159 cells identified in **(c)**. ** Indicates P<0.01, Mann-Whitney U-test. **(e)** Heatmap showing z-score scaled expression values for genes defining the two clusters depicted in **(c)**. Cluster 2, which is composed of cells with a significantly higher mass (P<0.01, Mann-Whitney U-test), shows increased expression of various genes relating to cell cycle progression.

Figure S10

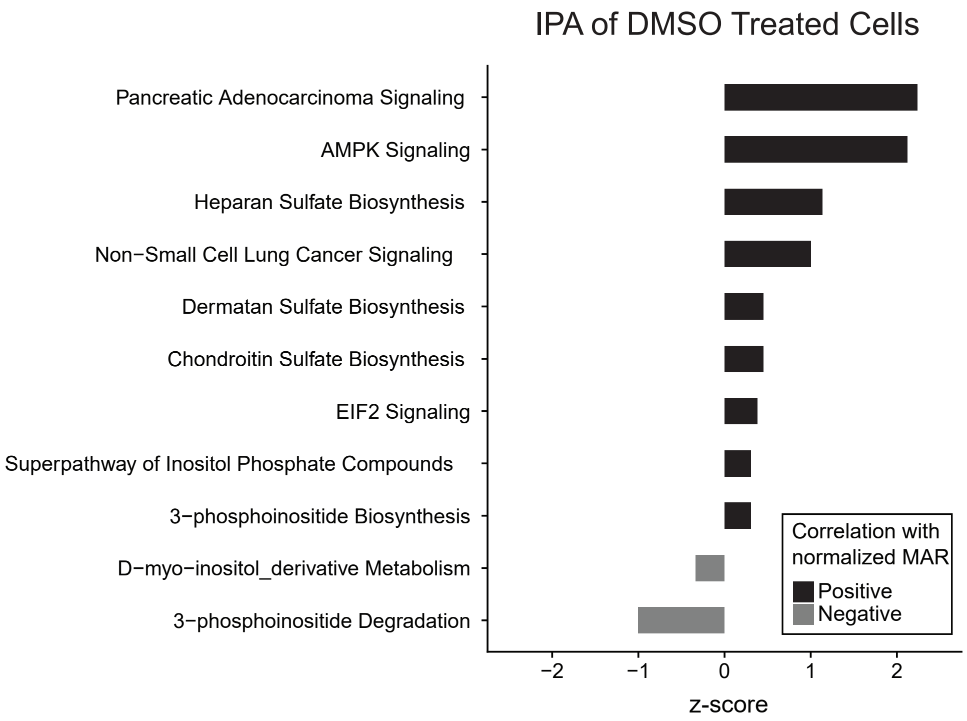

Figure S10 | IPA of DMSO treated BT159 cells

Plot of significantly enriched canonical pathways (FDR<0.05) in DMSO treated BT159 cells (n = 83), as determined by Ingenuity Pathway Analysis, amongst genes with significant positive (black) or negative (gray) correlations with normalized MAR. (**Additional File 1: Figure S4, Additional File 11: Table S10, Methods**).

Supplementary Notes

Note S1 | Maintaining minimum cell spacing in mass sensor array

Loading single cells into the mass sensor array at a fixed, minimum spacing requires the implementation of active switching between two distinct fluidic states. Initially, equivalent pressures are applied to the upstream and downstream ports on the bypass channel leading in to the array (**Additional File 1: Figure S1**, ports P1 and P3). In this "loading" configuration, all streamlines are directed into the array and therefore cells in the bypass channel will enter the array. An imaging region at the entrance to the mass sensor array (outlined in **Additional File 1: Figure S1**) is used as an indication of when a cell has been successfully loaded. Real-time optical peak detection within this region is used to switch from this loading fluidic state to a "flushing" regime wherein the upstream pressures (P1) is increased and the downstream pressure (P3) is decreased such that a vast majority of streamlines continue along the bypass channel with a small fraction entering the array. Because cells are of finite size and occupy several streamlines, they are directed along the bypass channel and not drawn in to the array. Importantly, during this process the pressure at the entrance to the mass sensor array is maintained at a fixed value, therefore any cells that have entered the array continue to flow at a constant speed. Therefore, although the volumetric flow rate is maintained across the array while flushing, no additional cells are loaded. After a desired amount of time has elapsed the system is automatically returned to the loading configuration to obtain the next cell for measurement.

Note S2 | Determining reproducibility of gene signatures related to mass and MAR

In order to determine the reliability and reproducibility of the linked biophysical and gene expression profiles, it was important to compare these signatures with additional results collected from independent experiments. For L1210 cells, single-cell gene expression profiles had previously been collected for cells with known times since division (TSD), a proxy for cell cycle progression [25]. We therefore hypothesized that the list of genes with expression levels that correlated significantly with single-cell mass (an alternative proxy for cell cycle progression) would show significant overlap with genes that correlated strongly with TSD. To determine the extent of this similarity, we constructed two test gene sets for gene set enrichment analyses: one which included genes with a significant positive correlation with cell mass and another which included genes with a significant negative correlation with cell mass (**Additional File 1: Figure S6a, Additional File 2: Table S1**). These gene subsets were compared to the full L1210 gene list measured previously, with genes ranked by how strongly their expression levels correlated with TSD. Genes with a significant positive correlation with mass were significantly over-represented amongst genes that showed a positive correlation with TSD in prior measurements (FDR<0.05). Similarly, genes with a significant negative correlation with mass were significantly over-represented amongst genes that showed a negative correlation with TSD (FDR<0.05). These results indicate that similar sets of genes are correlated with both TSD and single-cell mass, suggesting consistency between the measurements collected here and those collected previously.

Next, we sought to perform a similar comparison for FL5.12 cells. However, in contrast to L1210 cells, no single-cell gene expression measurements had been collected for these cells previously. We therefore conducted a second, independent experiment where single-cell mass and MAR measurements were

collected upstream of scRNA-seq for FL5.12 cells (**Additional File 1: Figure S6b,c**). Using this independent data set, we generated full gene lists that were ranked by correlation strength with either mass or mass-normalized MAR. Then we once again constructed test gene sets, this time containing genes from the original FL5.12 data set with significant correlations (both positive and negative) with either mass or mass-normalized MAR ($P<0.05$). Following the same analysis described above, we found that gene sets correlating with both mass and mass-normalized MAR showed significant overlap between both replicate experiments ($FDR<0.05$). This once again demonstrates the reproducibility of the gene expression signatures that correlate with single-cell biophysical properties.

Note S3 | Additional cell cycle gene expression analysis

To further validate the cell cycle-related gene expression signatures that correlated with cell mass, we performed an additional set of analyses relying on single-cell gene expression data alone modelled after the cell cycle interpretation approaches presented by Macosko et al. and Kowalczyk et al. [2, 24]. Briefly, we utilized gene lists found to be associated with the G1/S and G2/M stages of the cell cycle reported by Whitfield et al. [53]. Rough phase-specific scores were determined by calculating the average expression value (ln(TPM+1)) of genes from these lists that were detected in the data set of interest (L1210, FL5.12, or CD8+ T cells). The lists used for each phase were then filtered to only include genes that correlated strongly ($R>0.3$) with these rough scores. The average expression values across these remaining genes were then mean centered and divided by their standard deviation to yield the final G1/S and G2/M phase-specific scores. Each individual cell was assigned to either the G1/S or G2/M phases based on which gene list yielded the maximal score. For all cell types, the mass of cells assigned to the G2/M phase were significantly greater than those assigned to the G1/S phase ($P<0.001$, Mann-Whitney U Test, boxplots in **Additional File 1: Figure S5**). Furthermore, cells ranked by mass showed clear negative and positive relationships with G1/S and G2/M scores, respectively (heatmaps in **Additional File 1: Figure S5**). These results offer further evidence of a coordination between single-cell mass and cell cycle gene expression in L1210, FL5.12, and CD8+ T cells at various stages of activation.
